# Exploiting ovine immunology to improve the relevance of biomedical models

**DOI:** 10.1016/j.molimm.2014.09.002

**Published:** 2015-07

**Authors:** Gary Entrican, Sean R. Wattegedera, David J. Griffiths

**Affiliations:** Moredun Research Institute, Pentlands Science Park, Bush Loan, Edinburgh EH26 0PZ, Scotland, UK

**Keywords:** Sheep, Biomedical models, Immunology, Reproductive disease, Lung disease

## Abstract

Animal models of human disease are important tools in many areas of biomedicine; for example, in infectious disease research and in the development of novel drugs and medical devices. Most studies involving animals use rodents, in particular congenic mice, due to the availability of a wide number of strains and the ease with which they can be genetically manipulated. The use of mouse models has led to major advances in many fields of research, in particular in immunology but despite these advances, no animal model can exactly reproduce all the features of human disease. It is increasingly becoming recognised that in many circumstances mice do not provide the best model and that alternative species may be more appropriate. Here, we describe the relative merits of sheep as biomedical models for human physiology and disease in comparison to mice, with a particular focus on reproductive and respiratory pathogens.

## Highlights

• Sheep make a valuable contribution to immunology research.
• Lessons to be learned from studying infections in the natural host.
• Factors to consider when selecting biomedical models.

## 1 Introduction: Applications of animal models

The development of biomedical products (vaccines, chemotherapeutics, and devices) requires a deep functional understanding of how they exert their effects and perform over time, with safety being a primary criterion. *In vitro* cell culture systems provide a wealth of functional information on many aspects of cell activation, proliferation and controlled cell death. However, there is currently no *in vitro* substitute for *in vivo* experimentation that provides insight into the complex interactions of multiple cell types within organ structures. This is particularly true for the immune system that depends on cells migrating between and within anatomical compartments to reach defined areas of organised lymphoid tissues to become activated and then migrate back to the periphery to exert their effects. Consequently, it is likely that animal models will remain an essential component for the safe and effective translation of biological products and devices that interact with the immune system for applications in both human and animal medicine.

Rodents (mice, rats, guinea pigs) and lagomorphs (rabbits) have traditionally been the species of choice for biomedical models of human disease, the application of which has resulted in enormous advances in medicine. The validity of any animal model for determining biological effects in another species is always open to debate; hence the choice of model should mimic the biological effect in question as closely as possible for the target species. There are multiple criteria that influence this choice which often means that some features need to be balanced off against others, this will depend on the hypothesis being tested. The size, physiology, immunology and temperament of animals as well as the practical means to conduct the experiments (infrastructure facilities, tools and reagents) are all factors in the selection of animal models. Any one feature of a model that is desirable for one purpose may be a distinct disadvantage for another. In this review, we will discuss the pros and cons of sheep as biomedical models and discuss prospects for the future.

It is worth stating at the outset that although animal models are essential for understanding integrated biological systems, their use is tightly regulated and alternative methods to animal experimentation are employed wherever possible. There are a number of bodies globally that promote alternative means of conducting biological research that does not involve animals. These include the UK National Centre for the Replacement, Refinement and Reduction (NC3Rs) of Animals in Research (URL 1), the European Union Reference Laboratory for Alternatives to Animal Testing (EURL ECVAM) which aims to reduce, refine or replace the use of animals for safety and efficacy testing of chemicals, biologicals and vaccines (URL2) and in the United States there is the Interagency Coordinating Committee on the Validation of Alternative Methods (ICCVAM) which establishes guidelines, recommendations, and regulations that promote the regulatory acceptance of valid tests while reducing, refining, or replacing animal tests and ensuring human safety and product effectiveness (URL3). A recent workshop recommended that international harmonization and collaboration between human and veterinary researchers would accelerate progress on the 3Rs. This is particularly important when considering the validation and application of animal models for human disease (Stokes et al., 2012).

Although the principles of the 3Rs are becoming more deeply embedded in research, the benefit of animal research for human health is (quite rightly) open to debate and challenge and *in vitro/in silico* alternatives should be sought wherever possible. There is no doubt, however, that there are many major medical advances that would not have been made without animal models (Matthews, 2008). Ten years ago, it was proposed that there should be more systematic reviews of animal studies as this would inform more robust experimental design and ensure that research outcomes are translated to clinical benefits more effectively (Pound and Bracken, 2014). Central to this debate is the predictive value of animal models and the available alternatives for human medicine. Inter-species differences in genetics, epigenetics and physiology can all potentially influence the performance of biomedical products and therefore studies in one species may not predict performance in another. The predictive value of certain models, particularly those relating to human drug development, is still questioned and regarded as sub-optimal by some (Pound and Bracken, 2014). However, considering the cellular and molecular interactions involved in immune activation, *in vitro* observations using cell and organ culture can only go so far and cannot replicate or predict parameters such as the magnitude, quality and duration of immunity induced by vaccines. The approach therefore should be the selection of the most appropriate model available. To date, laboratory mice have been the model of choice for most immunology studies as they can be easily produced, handled and genetically manipulated. They may not always be the most appropriate model but may be the best that is available. A wider range of models to choose from should therefore ultimately reduce animal usage as the most relevant would be used for any particular given purpose.

## 2 Sheep as biomedical models

No animal model will exactly mimic human disease or precisely reproduce the effects of prophylactic/therapeutic agents or medical devices that are developed for human use. The choice of model depends on many factors and is dictated by the nature of the investigation/product and the relative advantages and disadvantages of each available model (Ducrot et al., 2011). Sheep are large and lend themselves to longitudinal analyses and repeat sampling from individual animals over time from a variety of anatomical compartments such as blood, lymph, and lung. Their size is a particular advantage for physiological models such as respiratory function, cardiovascular/ischemic disease, orthopaedics and reproductive/pregnancy-related disorders. They are also outbred and therefore representative of population diversity. All of these features are in contrast to the traditional small animal congenic mouse models. By comparison, mice have advantages in that they can be easily genetically manipulated and consequently have provided a wealth of mechanistic knowledge, particularly in relation to basic immunology.

There have been many published articles and reviews of sheep as models for a variety of human diseases/disorders and prophylactics/treatments. These include pregnancy disorders (Barry and Anthony, 2008), intrauterine inflammation (Melville et al., 2012; Collins et al., 2013), osteoporosis (Oheim et al., 2012), osteoarthritis (Gregory et al., 2012) respiratory syncytial virus (RSV) infection (Derscheid and Ackermann, 2012), asthma (Meeusen et al., 2009), vaccination (Scheerlinck et al., 2008), *in utero* gene therapy (Mehta et al., 2012), bacterial lung infection (Collie et al., 2013), acute lung injury and respiratory distress syndrome (Fernandez-Bustamante et al., 2012; Ballard-Croft et al., 2012), airway epithelial repair (Yahaya, 2012), preterm bronchopulmonary dysplasia (Albertine, 2013), aortic valve replacement (Martin and Sun, 2012) and stem cell therapy (Harding et al., 2013). In some cases sheep have been found to be a good model for humans (lung disease) but not for others (aortic valve replacement). It is not the purpose of this review to repeat the contents of these previously published reviews, particularly the physiological models, although we will refer to them. Here we will focus more on the immunological aspects of sheep in relation to models of disease and human disorders. To do that we firstly need to consider our capability to dissect and analyse the ovine immune system.

### 2.1 Identification of immunological parameters in sheep: cell subsets

Knowledge of the architecture of the immune system and the capability to measure the appropriate immunological parameters are prerequisites for validating *in vivo* models that involve immune activation and inflammation. This applies to models that investigate infectious diseases, models that evaluate vaccines and vaccine platforms and models that test orthopaedic medical devices.

Immune activation in mammals occurs through a complex series of molecular and cellular events that involves cell migration from blood and tissues to organised areas of lymph nodes where lymphoid and myeloid cells can interact in close proximity (Forster et al., 2012). Sheep have been a fundamental model for the elucidation of these processes. It is almost 30 years since a portfolio of monoclonal antibodies (Mabs) was produced to identify lymphoid cell subsets in sheep. These Mabs were fundamental to the ground-breaking studies that defined lymphoid cell subset distribution and recirculation between the blood and lymph (Mackay et al., 1985; Maddox et al., 1985; Hein and Griebel, 2003). The ability to perform lymphatic cannulation and collect cells from both efferent and pseudo-afferent lymph from peripheral sites in sheep has given major insights into immune activation and cell migration that could not be done physically in small laboratory rodents. Furthermore, cannulated sheep can be maintained for long periods (over a week) which allows for longitudinal analyses of the dynamics of immune activation in individual animals (Hein and Griebel, 2003).

One of the early discoveries using these Mabs to phenotype cell subsets in sheep was the proportion of cells expressing the T19 molecule (Mackay et al., 1989). This molecule is now known as WC1 and identifies γδT cells (Lund et al., 1993). It became clear that sheep (particularly lambs) have a very high proportion of circulating γδT cells compared to humans and mice (Mackay et al., 1989; Baldwin et al., 2014). The genes encoding γ and δ T cell receptor chains do not exhibit the high variability seen in the genes encoding α and β T cell receptor chains and γδT cells may therefore act as a bridge between innate and adaptive immunity (Hein and Griebel, 2003). The exact functional significance of this variance in γδT cells between species remains unknown but may reflect the requirement for ruminants to respond rapidly to certain pathogen challenges compared to humans and mice. Nevertheless, this difference should be considered when analysing immune activation and effector mechanisms in situations where sheep are being used for comparative immunological studies as biomedical models.

Those first studies on the compartmentalisation and activation status of cell subsets in the lymph and blood of sheep focussed predominantly on B cells and T cells as there were few Mabs at that time to reliably define myeloid cells. The studies were also conducted using single-colour flow cytometry. It is now possible to conduct multicolour flow cytometry as a wider range of Mabs against ovine cell surface-expressed molecules has become available against both lymphoid and myeloid cell subsets (Pascale et al., 2008). Multicolour staining has revealed the complexity of dendritic cell (DC) subsets in humans and mice using a range of Mabs that react with CD8α, CD11b, CD11c, CD14, CD103, CD172a/SIRPα and CD207 among others (Guilliams et al., 2010). Mabs that react with the sheep orthologues of most of these molecules are also now available and will help elucidate DC subset distribution and function that are important for comparative species analyses. For example, the ability to identify, define and compare DC subsets in humans, mice and sheep might help to explain the difference in success rate of DNA vaccination in mice compared to sheep and hence inform on vaccine adjuvant and delivery platforms for translation to human medicine (Scheerlinck et al., 2008).

Another subset that until recently has been elusive and difficult to identify in sheep is natural killer (NK) cells. NK cells provide a very important first line of host defence against infection and tumours and are also found in abundance in decidualised mammalian placentation (Jabrane-Ferrat and Siewiera, 2014). The first detailed characterisation of putative NK cells in sheep identified a population of CD16^+ve^/CD14^-ve^/perforin^+ve^ cells in peripheral blood (Elhmouzi-Younes et al., 2010). It was impossible at that time to conduct the more characteristic NK phenotyping (CD335^+ve^/CD3^-ve^) due to the lack of Mabs against these molecules in sheep. Since then, a Mab has been produced against ovine NKp46 (CD335) using a cloning, expression and immunisation approach. This Mab works extremely well in flow cytometry and has been used to definitively define ovine NK cells (Connelley et al., 2011). This is a major advancement as it allows detailed investigation of innate immune activation in sheep for disease pathogenesis and vaccine studies as well as comparative reproductive biology models (see below). Interestingly, no-one has yet managed to produce a Mab that detects surface-expressed CD3 in sheep despite many attempts.

### 2.2 Identification of immunological parameters in sheep: cytokines and chemokines

There are a large number of Mabs that react with ovine cytokines/chemokines, several of which were developed against cattle but cross-react with the sheep orthologue. The major commercial sources of immunological reagents for sheep have been described previously (Entrican and Lunney, 2012). These Mabs can be used in various formats to detect cytokine/chemokine expression (ELISA, ELISpot, intracellular cytokine staining). In addition to disease studies (see section 3.4), cytokines can be used as biomarkers/immune correlates for the osteolysis associated with debris following orthopaedic device implantation. For such studies, sheep have a practical advantage over small laboratory animals for the evaluation of human orthopaedic devices due to their size (Gregory et al., 2012). However, the relatively narrow range of cytokines that can be analysed in sheep is a barrier to understanding the reasons for device failure (URL4). As an example, the measurement of ovine IL-2, a cytokine fundamental to T cell activation, still remains problematic due to a lack of Mabs.

Multiplexing for the simultaneous detection of multiple cytokines/chemokines is routine in mice and humans. Given that ‘signatures’ of combined cytokine expression are much more informative than measurements of any one single cytokine, this is an area that needs to be addressed for ovine immunology and for the improvement of sheep models. One of the major barriers to research in sheep is the lack of a publically-accessible searchable database that lists the sources and functional characteristics of Mabs, recombinant cytokines and molecular probes for ovine immunology. The development, population and long-term curation of such a database requires a significant financial and time commitment which to date has been elusive to secure.

### 2.3 Identification of immune correlates in sheep

While certain factors can be controlled when designing experiments (age, gender, prior-exposure to defined pathogens), sheep are naturally outbred and therefore genetically diverse. They are also often maintained in open pens or fields which can result in exposure to a variety of environmental elements (and infection) not present in the highly controlled and regulated accommodation used for maintaining small laboratory rodents. This diversity can be useful, for example to get a true idea of the effect of a treatment or vaccine in a population. Nevertheless, as all of these factors can introduce variation, group sizes need to be carefully considered when embarking on sheep experiments to ensure that meaningful data can be generated. This is very important when measuring immunological parameters as there is clear evidence of not only *inter*-sheep variation but also of *intra*-sheep variation in longitudinal studies (Wattegedera et al., 2004).

Repeat analyses of sheep for *in vitro* correlates of immune activation has revealed the degree of variation that can be expected for mitogen-induced cell proliferation and cytokine production from the same animals over time. In contrast to mice and other small laboratory rodents, large numbers of peripheral blood mononuclear cells (PBMC) are easily recoverable from sheep by venipuncture and are therefore commonly used as an experimental readout. This is a particularly useful source of material for longitudinal analyses of antigen-specific cellular immune recall responses. A study on 24 normal female sheep revealed a ten-fold difference in their PBMC responses to *in vitro* stimulation with the T cell mitogen Concanavalin A, as measured by cell proliferation and interferon (IFN)-γ production (Wattegedera et al., 2004). Longitudinal analyses of the same sheep over several weeks showed that the highest responders at the first sample point were not the highest responders at the second or third sample point for the same animals. Furthermore, there was not a clear correlation between cell proliferation and IFN-γ production. These results show the importance of using group sizes that have sufficient statistical power when designing experiments involving sheep (or other outbred animals). They also demonstrate that the choice of read-out needs to be carefully considered for measurement of cellular immune function for the model in question.

### 2.4 Infection studies in sheep

There is a multitude of *in vivo* experimental infection models of diseases that are of concern to the sheep farming industry. These models closely mimic the host–pathogen interactions that occur in the natural host and are therefore of great value and importance. It is not within the scope of this review to cover all of those sheep diseases but to focus on those that have biomedical relevance for humans. One viewpoint is that the models most likely to represent human disease would be zoonotic pathogens that naturally infect both sheep and humans, of which there are many examples. An alternative viewpoint is that host-adapted strains of pathogens might be more representative of disease progression in their specific host and therefore be better models. We will discuss the value of such models using respiratory syncytial virus (RSV), *Chlamydia abortus* and retroviruses as examples.

#### 2.4.1 Respiratory syncytial virus

Respiratory syncytial virus (RSV) is an important respiratory pathogen that causes significant morbidity and mortality, particularly in neonatal infants (Tregoning and Schwarze, 2010). Sheep provide a valuable model to study infection with RSV as they are susceptible to ovine, bovine and human strains of the virus and therefore allow directly comparative studies (reviewed in detail by Derscheid and Ackermann, 2012 and Bem et al., 2011). In particular, an experimental model of human RSV (hRSV) infection has been established in pre-term and neonatal lambs (Olivier et al., 2009; Sow et al., 2011). Infection of lambs using this model results in mild to moderate bronchiolitis that replicates many of the features of RSV pathology in young infants, including injury to bronchiolar and alveolar epithelial cells, activation of pro-inflammatory cytokines and chemokines, and recruitment of neutrophils, macrophages and lymphocytes. In this model, lambs may be infected by intratracheal or intrasnasal injection or by use of a nebulising inhaler to mimic natural infection (Derscheid and Ackermann, 2012; Bem et al., 2011). An important strength of the ovine model for hRSV is the ability to produce pre-term lambs by caesarean as this provides a better model of RSV infection in premature babies. This reflects the closer pattern of alveolarisation of sheep and human lungs at birth, in comparison to rodent lungs (Derscheid and Ackermann, 2012).

Most studies of RSV in lambs have utilised the common laboratory A2 strain of hRSV, which results in a mild form of pathology but other clinical strains can be used to produce more severe disease (Derscheid et al., 2014). The lamb model has been used to study the dynamics of virus replication and cytokine production (Olivier et al., 2009; Sow et al., 2011) and to examine the outcome of therapeutic interventions, including vaccine response. In this regard it is notable that a formalin-inactivated hRSV vaccine that exacerbates disease in infants also increased inflammatory damage in lambs (Derscheid et al., 2013). This suggests that the ovine model may be valuable for assessing future RSV vaccines prior to their use in children.

#### 2.4.2 Chlamydial infection

The genus *Chlamydia* comprises nine species of obligate intracellular Gram-negative bacteria that infect a wide variety of hosts including humans, mice and sheep (Horn, 2011). *Chlamydia abortus* is a major cause of abortion in sheep, resulting in substantial lamb production losses in most sheep-rearing countries worldwide (Longbottom et al., 2013a). Abortion almost always occurs in late gestation and is due to severe placentitis characterised by pro-inflammatory cytokine (TNF-α) and chemokine (CXCL8) production, inflammatory cell infiltration and local thrombosis that starves the foetus of nutrients and gaseous exchange (Entrican et al., 2010). The strongest immune correlate for protection against chlamydial abortion in sheep is IFN-γ (Entrican et al., 2012). *C. abortus* is also a zoonotic pathogen which can cause abortion in pregnant women (Buxton, 1986). Sheep might therefore be considered a good model for understanding human infection with *C. abortus* but this is not the case due to some very notable differences in disease pathogenesis between the two hosts. Firstly, sheep that are infected before pregnancy can harbour the organism in a persistent subclinical manner that only manifests itself in the subsequent pregnancy. The site of this persistence is unknown (Longbottom et al., 2013b). In contrast, there is no evidence for persistence in humans as all clinical cases of abortion in women have been linked to direct exposure to contaminated material during pregnancy (Eddy and Martin, 1986). Secondly, pregnant ewes show few signs that they are about to abort, do not require treatment such as antibiotics and after abortion will return to normal health very quickly. In contrast, infection in pregnant women is clinically severe, manifested by disseminated intravascular coagulation which can be fatal if not immediately treated (Buxton, 1986; Eddy and Martin, 1986). This is an extreme outcome and is one of the few situations where chlamydial infection is rapidly fatal in any host. These differences between sheep and humans may be due in part to innate immune activation by the infection that prevents persistence being established in humans. They may also be reflective of the different placental structures in sheep and humans and mechanisms of immune modulation at the materno–foetal interface. What this does show is that the same pathogen can behave very differently in two hosts and there are lessons to be learned from host–pathogen adaptations. In the case of *C. abortus* it is the mechanisms by which it persists and ultimately causes disease that could be informative for chlamydial–host interactions in other species including humans. It should be noted that there are mouse models of *C. abortus*-induced abortion but as for humans there is no evidence of persistence and the models are used primarily as a rapid method of screening potential vaccines to protect against abortion in sheep and not as models for human infection (Kerr et al., 2005).

**Fig. 1:**
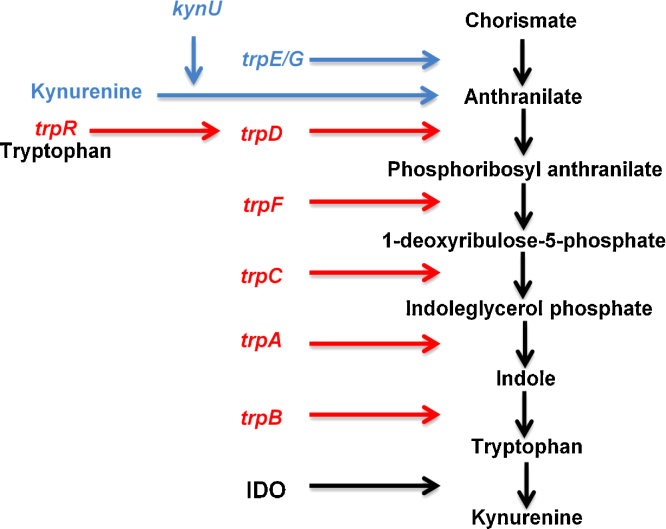
The tryptophan biosynthetic pathway. Tryptophan is synthesised from chorismate and kynurenine via anthranilate. The chorismate to anthranilate step is mediated by *trpE/G* (genes are present in *Escherichia coli* but not present in *Chlamydia spp.*) whereas the kynurenine to anthranilate step is mediated by host *kynurinase*. *Chlamydia pecorum* and *Chlamydia caviae* encode an almost complete tryptophan biosynthesis operon that includes the tryptophan repressor (*trpRDCFBA*) and is switched on when intracellular tryptophan levels decrease. *Chlamydia abortus* and *Chlamydia muridarum* lack all of these genes*, Chlamydia trachomatis* encodes only *trpRBA*. Tryptophan is degraded by the host enzyme indoleamine 2,3-dioxygenase (IDO) which can be induced by interferon-γ or constitutively expressed, most notably by foetal trophoblasts in the placenta. Adapted from Wood et al. (2004).

The major chlamydial pathogens of humans are *C. trachomatis* (genital/ocular infection) and *C. pneumoniae* (pulmonary infection). Neither of these species is known to be a natural pathogen of sheep or mice, although *C. pneumoniae* does have a very wide host range. Mice are the biomedical models of choice for developing human vaccines against *C. trachomatis*, although *C. muridarum*, a mouse pathogen that is phylogenetically closely-related to *C. trachomatis*, is also used as a biomedical disease model and may be more appropriate as it represents a more natural host–pathogen interaction. As for *C. abortus* infection in sheep, IFN-γ production is a very strong immune correlate of protection against chlamydial infection in humans and mice (Rottenberg et al., 2002). Chlamydiae are well-adapted to their natural hosts and also to their preferred anatomical niche within that host (Borges et al., 2012; Abdelsamed et al., 2013). This adaption includes the ability to persist intracellularly without killing the host cell or the host cell being killed by the immune system. Chlamydiae rely on their hosts for many nutrients, which includes amino acids such as tryptophan (Trp). Trp is an essential amino acid for mammals and is synthesised from chorismate or kynurenine via anthranilate by tryptophan synthase in plants and micro-organisms (Fig. 1). Trp can be degraded by the enzyme indoleamine 2,3-dioxygenase (IDO) which is inducible by IFN-γ. Therefore, dependency on host cell Trp for growth by Chlamydiae can make them vulnerable to the host IFN-γ response. The *C. abortus* genome lacks all of the components of the tryptophan biosynthesis operon (Thomson et al., 2005) whereas the related sheep pathogen, *C. pecorum* has an almost complete tryptophan biosynthesis operon that encodes the genes for *trpRDCFBA* (Sait et al., 2014). This suggests that *C. pecorum* can synthesise Trp from anthranilate whereas *C. abortus* cannot (Fig. 1), potentially resulting in immune evasion. *C. trachomatis* can also evade the IFN-γ/IDO pathway in human epithelial cells by switching on Trp biosynthesis whereas *C. muridarum* does not (Nelson et al., 2005). In contrast, *C. trachomatis* is very susceptible to IFN-γ-mediated restriction in murine cells due to immunity-related GTPases whereas *C. muridarum* has evolved to evade this IFN-γ-induced pathway (Coers et al., 2008). Collectively, these observations show that great care needs to be taken when comparing *in vitro* and *in vivo* models of not just chlamydial infections but to other pathogens that are controlled by IFN-γ (Coers et al., 2009). Thus, the sheep experimental models of *C. abortus* are excellent for replicating disease and abortion and this has direct relevance for sheep, but is not a representative model for human chlamydial abortion. Additionally, there is no clear evidence to suggest that sheep would be a good biomedical model for either *C. trachomatis* genital or ocular infection in humans even though both hosts share the IFN-γ-induced IDO defence pathway.

#### 2.4.3 Retroviruses

Retroviruses are important pathogens of many species, including sheep, and are associated with a wide range of diseases, including immunological and neurological disease and cancer. The study of retroviral diseases in animals has provided a wealth of information that has greatly contributed to our understanding of the pathogenesis of human diseases. Perhaps the most significant contribution has been studies on oncogenic animal retroviruses that led to the discovery of cellular proto-oncogenes, a concept that underlies much of our understanding of the molecular basis of cancer (Vogt, 2012). Two retroviruses of sheep are of particular interest with respect to model systems; jaagsiekte sheep retrovirus (JSRV) which causes ovine pulmonary adenocarcinoma (OPA) (Griffiths et al., 2010; Palmarini and Fan, 2001) and visna/maedi virus (VMV), which causes inflammatory disease in a number of tissues, particularly lung and the central nervous system (Thormar, 2005). Both of these are common and significant problems for sheep farmers, but they are also recognised as valuable animal models for human disease.

OPA shares histological similarities with some forms of human lung adenocarcinoma, particularly subtypes of the tumour that are relatively non-invasive and only weakly associated with tobacco smoking (Sun et al., 2007). While JSRV is not thought to infect humans, it is possible that the oncogenic mechanisms involved in the two species may have some similarity. JSRV is unusual among oncogenic retroviruses in that it does not appear to use insertional activation of oncogenes as a transformation mechanism. Instead, JSRV carries its own oncogene, which triggers neoplastic transformation through the activation of cellular protein kinase signalling pathways (Maeda et al., 2008). Notably, an experimental disease model utilising neonatal lambs has been established in which tumours and associated respiratory distress are evident within several weeks of infection with JSRV (Martin et al., 1976; Palmarini et al., 1999). In addition, recent studies have shown that small JSRV-infected foci comprising single cells or small numbers of cells can be detected in the lung 10 days after experimental infection (Martineau et al., 2011; Murgia et al., 2011). This model therefore provides an opportunity for longitudinal studies of lung cancer pathogenesis beginning with a single transformed cell through to the development of large tumours. To date, this model has mainly been used to examine aspects of virus–host interaction in OPA (Caporale et al., 2006; Cousens et al., 2007; Caporale et al., 2013) but it shows great promise as a platform for future studies on lung cancer pathogenesis; for example, in developing *in vivo* cancer imaging methods or studying early events in tumourigenesis. This is particularly important given the poor prognosis of human lung cancer and the difficulty of studying early tumours in humans.

OPA is also of interest for studying the immunological aspects of host–virus and host–tumour interactions. Natural infection by JSRV is characterised by the lack of a significant adaptive immune response to JSRV proteins. This is thought to be due to the expression of endogenous retroviruses (ERVs) in the sheep genome that are closely related to JSRV. ERVs are the remnants of ancient retroviruses that integrated into the germ-line of vertebrates during evolution (Gifford and Tristem, 2003). In most cases, ERVs integrated millions of years ago and the large majority are now defective and cannot produce infectious viruses. They can nevertheless contribute important functions to host physiology; for example, impacting on immune responses to infections and on placental development and function. In the case of endogenous JSRVs (denoted enJSRV), their expression in the foetal thymus has been proposed to result in central tolerance to antigens from the infectious, oncogenic, JSRV (Palmarini et al., 2004). However, there is good evidence that the sheep immune system can respond to JSRV antigens. For example, anti-JSRV antibodies and cellular responses to JSRV Gag (capsid) proteins have been described in sheep following administration of recombinant protein and adjuvant (Summers et al., 2006). In addition, lambs experimentally infected with JSRV can develop neutralising antibodies to JSRV that target the viral surface envelope (Env) glycoproteins (Hudachek et al., 2010; Caporale et al., 2013). These observations indicate that there are at least small populations of JSRV-reactive B and T cells present in sheep and therefore that the central tolerance hypothesis for lymphocyte clonal deletion cannot fully explain the pathogenesis of OPA. This presents JSRV/OPA as a model for studying host tolerance to retrovirus infection in a large outbred model.

A notable histological feature of OPA is the presence of abundant macrophages at the periphery of the tumour foci (Summers et al., 2005). These cells are found in experimentally-infected lambs as well as in natural cases of disease, indicating the presence of an innate cellular response to JSRV infection and/or to the growth of the tumour. Studies in a variety of human and murine cancers have shown that tumour associated macrophages (TAMs) may exhibit a wide spectrum of phenotypes and produce a variety of cytokines and chemokines that can promote, rather than inhibit, the growth and spread of tumours (Schmieder et al., 2012). In addition, tumour cells may themselves express factors that drive macrophages towards a ‘pro-tumour′ phenotype. Interestingly, the phenotype of macrophages can be highly variable even in different locations of the same tumour (Ruffell et al., 2012). Although mice have been a valuable model for studying macrophage polarisation in the context of tumours, it is recognised that there are many differences between the markers expressed by these cells in humans and mice (Mestas and Hughes, 2004). OPA therefore provides an additional natural tumour model in which to investigate the function of TAMs. Few studies have been performed to date, and the limited data available suggest that at least some OPA TAMs express IFN-γ which would suggest a pro-inflammatory, anti-tumour phenotype (Summers et al., 2005). However, more detailed analysis of these cells is required to fully assess their role in pathogenesis. It is possible that TAMs augment local immunosuppression in OPA and contribute to the lack of adaptive response to JSRV.

VMV is a lentivirus that was first characterised in the 1950s by Sigurdsson and colleagues (Sigurdsson et al., 1957; Thormar, 2013). This work was of huge significance in the development of the concept of ‘slow′ virus infections to describe diseases with a chronic, progressive course. VMV and related small ruminant lentiviruses have been regarded as an important large animal model for studying chronic inflammatory disease, in particular in the lung, joints and central nervous system (Narayan and Cork, 1985). In addition, VMV pathogenesis shares a number of features with HIV infection including tropism for macrophages, a slow disease progression, and a strong anti-viral immune response that drives rapid virus evolution and promotes immune escape. Studies on VMV and other non-primate lentiviruses in their natural hosts have therefore provided comparative data on lentiviral pathogenesis. For example, VMV and HIV share the ability to invade the central nervous system and VMV has therefore attracted attention as a model for lentivirus-induced encephalitis. More recently, VMV has been proposed as a valuable model for informing the development of vaccines to lentiviruses and in developing antiviral therapies (Thormar, 2013). However, there are some very important differences between VMV and HIV pathogenesis, the main one being that VMV does not infect lymphocytes and so immunosuppression due to loss of CD4+ T cells does not occur. Nevertheless, as for Chlamydia the value of studying retroviruses in their natural host is that this may be more informative of fundamental pathogenic mechanisms than using a human pathogen in a biomedical model.

### 2.5 Reproductive and respiratory models

As previously mentioned, sheep have been successfully used as biomedical models of pre-natal disorders and *in utero* therapies for humans, including immunological analyses of lymphoid cell populations, cytokines and chemokines that correlate with inflammation and tissue damage (Melville et al., 2012; Sadowska et al., 2012; Collins et al., 2013). Sheep are often considered better suited as models for human pregnancy disorders than mice due to their size and gestation period. However, there are some very notable physiological and immunological features of placentation and reproduction that are more closely shared between humans and mice than between humans and sheep (Table 1). The major physiological difference is in the structure and degree of invasiveness of the different placentas. Sheep have a cotyledonary, synepitheliochorial placenta (Entrican and Wheelhouse, 2006), whereas humans and mice have discoid, haemochorial placentation (Carter and Enders, 2004). In sheep the most intimate contact between mother and foetus occurs in the cotyledons, with foetal binucleate trophoblast cells fusing with maternal uterine epithelial cells to form trinucleate plaques. Therefore in sheep there is considerable separation of the foetal trophoblast from the maternal blood. In comparison in humans and mice, multiple foetal trophoblasts fuse to form large multinucleated cell layers (syncytiotrophoblasts) that constitute the surface of placental villi and are directly bathed in maternal blood. However, it should also be noted that the cotyledons of the sheep placenta contain interdigitating villi between the foetus and mother that share similarity with the foetal villi in humans (Barry and Anthony, 2008).

The differing patterns of cell-to-cell fusion that occur during placental morphogenesis are thought to be driven by ERVs (reviewed by Lavialle et al., 2013). The Env proteins of infectious retroviruses are involved in fusing virus and cell membranes during infection. This fusogenic activity has been retained in some ERVs and has been co-opted by their mammalian hosts to drive cell to cell fusion, resulting in syncytial cell formation in the placenta. The placentas of humans and mice have one or two syncytiotrophoblast cell layers, respectively. In the mouse, each fused cell layer requires the fusogenic activity of a distinct ERV Env protein, denoted syncytin-A and syncytin-B (Dupressoir et al., 2009; Dupressoir et al., 2011). Humans also encode two syncytin proteins, denoted syncytin-1 and syncytin-2, which both appear to be involved in syncytiotrophoblast formation (Vargas et al., 2009; Mi et al., 2000). Notably, despite their functional similarity the human and mouse syncytins are entirely distinct and represent independent integration of ERVs that were acquired by their hosts at different points in their evolutionary history. Importantly, experimental deletion of murine syncytins results in foetal death (Dupressoir et al., 2011) and dysregulation of human syncytins is associated with preeclampsia (Vargas et al., 2011), confirming that these proteins have important roles in maintaining healthy pregnancy.

In sheep, two groups of ERVs have been identified as having essential roles in the establishment of pregnancy and development of the sheep placenta. The first, denoted ‘syncytin-Rum1′ has been shown to mediate cell fusion *in vitro* and to be specifically expressed in foetal binucleate cells, strongly indicating that it drives the formation of trinucleate syncytial plaques in sheep and cows (Cornelis et al., 2013). The second ERV shown to have a role in pregnancy is enJSRV. Specific knockdown of enJSRV expression *in vivo* in the early blastocyst retards growth of the conceptus, reduces IFN-τ expression and results in pregnancy loss by day 20 (Dunlap et al., 2006). These experiments indicate that enJSRV is essential for establishing a successful pregnancy in sheep but whether this acts via cell fusion or by a different mechanism has not been determined.

These studies, together with work in lagomorphs and carnivores (Cornelis et al., 2012; Heidmann et al., 2009; Lavialle et al., 2013), have shown than at least five mammalian orders have independently acquired different ERVs with fusogenic activity. This has been proposed to account, at least in part, for the variety of placental structures found in different species (Cornelis et al., 2013). In addition, it has been suggested that the acquisition of fusogenic ERVs was instrumental in driving the emergence of placental mammals from egg laying animals (Lavialle et al., 2013). Thus, with regard to the use of sheep as a model species, in this case the comparative aspects of placental biology in sheep, mice and humans have collectively led to greater understanding of the processes underlying placental morphogenesis. Moreover, the ability to perform *in vivo* gene knockdowns in sheep, as well as in mice, promote sheep as a model species for future studies investigating placental development. Furthermore, human syncytin-1 has also been linked with other cellular processes involving multinucleated cells, including osteoclast and myoblast fusion during bone and skeletal muscle development (Soe et al., 2011; Bjerregard et al., 2014). Demonstration of similar roles for syncytin-Rum1 could provide a novel model for studying these processes in sheep.

The intimate relationship of maternal and foetal tissues in the placenta requires some degree of immunological modulation to allow maternal acceptance of the semi-allogeneic foetus. Multiple immunomodulatory mechanisms have been identified in the placenta, with most described in mice and humans (Table 1). One potential mechanism involves ERV syncytin proteins, some of which possess immunosuppressive properties in addition to their fusogenic activity (Mangeney et al., 2007). Sheep syncytin-Rum1 has a predicted immunosuppressive peptide although its activity has yet to be studied. Another mechanism is IDO expression. In contrast to the antimicrobial IFN-γ-inducible IDO expression described above, expression of IDO by human and murine foetal trophoblast is constitutive and has been shown, at least in mice, to tolerise maternal T cells in the placenta to paternal MHC class I (Munn et al., 1998; Honig et al., 2004). There are no publications describing expression of IDO in the sheep placenta and if it is present it raises a very interesting paradox: why would a pathogen such as *C. abortus* target an organ where the availability of Trp is limited (Entrican et al., 2009)? This remains to be resolved along with a number of other unanswered questions regarding the immunological status of the ovine placenta. Such knowledge would be very informative for selecting biomedical models of reproduction as there does not appear to be an exact match for humans using either mice or sheep (Table 1).

An aspect of reproduction where sheep do differ from humans and mice is the reported modulation of the maternal peripheral immune system. There is no evidence that pregnant sheep exhibit the widely-accepted paradigm of a bias shift in maternal immunity from a Th1-phenotype (IFN-γ production) to a Th2 phenotype (IL-4 production) (Wattegedera et al., 2008). This may be related to the non-invasive synepitheliochorial placentation of sheep compared to the invasive haemochorial placentation of humans and mice, which could result in different levels of maternal immune modulation to prevent foetal rejection (Table 1).

Sheep are also useful as biomedical models of respiratory disorders. The sheep lung has a number of characteristics that support its use as a model for studies of human pulmonary physiology and disease in preference to rodents (Scheerlinck et al., 2008). These include greater anatomical similarity with human lungs and closer similarity in the distribution of specialised airway epithelial cells. In addition, the similar size of sheep and human lungs facilitates analysis of pulmonary function and permits multiple sampling and *in vivo* imaging procedures. The ability to use regional bronchoscopic instillation in sheep provides additional flexibility in the use of sheep as a model species (Meeusen et al., 2009). This is particularly beneficial for infection studies where different segments of lung can be instilled with different treatments and compared within the same animal, thereby facilitating longitudinal studies of chronic infection (Collie et al., 2013).

## 3 Perspectives and conclusions

Sheep diverged from the common ancestor of humans and mice approximately 100 million years ago, while humans and mice diverged 90 million years ago (Jiang et al., 2014). Mice have been the standard biomedical model for humans but the immunological relevance of mice for humans has been questioned due to differences in a whole range of innate and adaptive immune parameters (Mestas and Hughes, 2004). It is currently impossible to draw any such comparisons with sheep as the range of probes, tools and reagents is nowhere near extensive enough, although the improving annotation of the sheep genome along with the ability to link genetic profiles with specific traits will advance this (Bidwell et al., 2014). A further difficulty for comparative immunology studies is the variation in immune-related genes which are among the most diverse in the genome (Barreiro and Quintana-Murci, 2010). This is not a surprise given that pathogens are major drivers of evolution. Comparative gene expression profiles of human and mouse monocytes has revealed some evolutionarily-conserved patterns but also sufficient differences to exhibit caution when using mice as biomedical models of human disease (Ingersoll et al., 2010). Ultimately the question for comparative immunology and use of models is how these genetic differences translate into functional differences. Functionally-conserved CD8α-like DC have been described in humans, mice and sheep indicating that vaccine delivery strategies could be evaluated across these species as biomedical models with careful interpretation (Contreras et al., 2010).

There have been several studies looking for associations between genes and disease resistance in sheep. A system's genetic approach has been adopted to try to identify genes from quantitative loci traits (QTL) associated with parasite resistance from data collected in five species (sheep, cattle, mice, rats, humans). The barrier to identifying the individual genes within those QTLs for sheep is the relatively poor annotation of the sheep genome, as mentioned above (Sayre and Harris, 2012). Polymorphisms in ovine cytokine genes (notably IFN-γ) have been associated with reproductive failure in sheep, although direct links to specific infectious diseases have not been established (Darlay et al., 2011). Ideally, genotype should be linked to immunological phenotype and although these polymorphisms have been identified, how they translate to immune function remains unclear.

Finally, one of the major advantages of using mice for biomedical studies is that they can be genetically manipulated. Furthermore, the use of congenic mice permits transfer of cells and tissues between individuals without immunological rejection. It should not be forgotten that sheep were the first mammals to be cloned from an adult somatic cell by nuclear transfer with the birth of Dolly (Wilmut et al., 1997). This was followed by the production of transgenic sheep (Colman, 1999). The use of transgenic technologies for sheep is not as advanced as in mice but has become more established in recent years, while methods for gene delivery to the lung in live sheep have also been developed (Griesenbach et al., 2011). The recent advent of methodologies for targeted gene disruption has opened up new possibilities for generating transgenic sheep in the future. While it is also true that studies in sheep have been somewhat hindered by the relatively limited availability of reagents, recent progress towards an annotated sheep genome sequence will help redress this issue in future studies.

## Conflicts of interest

GE and SW have a research agreement with BioRad AbD Serotec and receive royalties from the sale of ruminant immunological reagents marketed by BioRad AbD Serotec.

## Figures and Tables

**Table 1:** Comparative immunological features of reproduction in human, mouse and sheep.

Feature	Human	Mouse	Sheep	Reference
Gestation period	9 months	3 weeks	5 months	(Bainbridge, 2000;Entrican and Wheelhouse, 2006)
Typical number of fetuses per gestation	1	5–9	1–2	URL5
Type of placentation	Haemochorial, discoid	Haemochorial, discoid	Synepitheliochorial, cotyledonary	(Carter and Enders, 2004)
Uterine NK cells	Yes	Yes	?	(Croy et al., 2010)
Transfer of maternal antibody to the foetus	Yes	Yes	No	(Cervenak and Kacskovics, 2009)
Trophoblast expression of classical MHC Class I	Yes*	Yes	?	(Madeja et al., 2011)
Trophoblast expression of nonclassical MHC Class I	Yes	No	?	(Madeja et al., 2011)
Trophoblast expression of MHC Class II	No	No	?	(Murphy and Tomasi, 1998)
Trophoblast expression of IDO	Yes	Yes	?	(Munn and Mellor, 2013)
Alternation in maternal peripheral Th1/Th2 responsiveness**	Yes	Yes	No	(Wattegedera et al., 2008)

*Expression of classical MHC Class I in human trophoblast is restricted to HLA-C which has low polymorphism compared to HLA-A and HLA-B. **Maternal immunity becomes biased towards a Th2 response and away from a Th1 response during gestation.
